# Development of the Korean Adult Reading Test (KART) to estimate premorbid intelligence in dementia patients

**DOI:** 10.1371/journal.pone.0181523

**Published:** 2017-07-19

**Authors:** Dahyun Yi, Eun Hyun Seo, Ji Young Han, Bo Kyung Sohn, Min Soo Byun, Jun Ho Lee, Young Min Choe, Suzy Ahn, Jong Inn Woo, Jongho Jun, Dong Young Lee

**Affiliations:** 1 Department of Neuropsychiatry, Seoul National University Hospital, Seoul, South Korea; 2 Premedical Science, College of Medicine, Chosun University, Gwangju, South Korea; 3 Department of Neuropsychiatry, Seoul Metropolitan Government-Seoul National University Boramae Medical Center, Seoul, South Korea; 4 Department of Neuropsychiatry, Ulsan University Hospital, Ulsan, South Korea; 5 Department of Linguistic, New York University, New York, NY, United States of America; 6 Department of Linguistic, Seoul National University, Seoul, South Korea; Istituto Di Ricerche Farmacologiche Mario Negri, ITALY

## Abstract

We aimed to develop a word-reading test for Korean-speaking adults using irregularly pronounced words that would be useful for estimation of premorbid intelligence. A linguist who specialized in Korean phonology selected 94 words that have irregular relationship between orthography and phonology. Sixty cognitively normal elderly (CN) and 31 patients with Alzheimer’s disease (AD) were asked to read out loud the words and were administered the Wechsler Adult Intelligence Scale, 4^th^ edition, Korean version (K-WAIS-IV). Among the 94 words, 50 words that did not show a significant difference between the CN and the AD group were selected and constituted the KART. Using the 30 CN calculation group (CNc), a linear regression equation was obtained in which the observed full-scale IQ (FSIQ) was regressed on the reading errors of the KART, where education was included as an additional variable. When the regressed equation computed from the CNc was applied to 30 CN individuals of the validation group (CNv), the predicted FSIQ adequately fit the observed FSIQ (*R*^*2*^ = 0.63). In addition, independent sample *t*-test showed that the KART-predicted IQs were not significantly different between the CNv and AD groups, whereas the performance of the AD group was significantly worse in the observed IQs. In addition, an extended validation of the KART was performed with a separate sample consisted of 84 CN, 56 elderly with mild cognitive impairment (MCI), and 43 AD patients who were administered comprehensive neuropsychological assessments in addition to the KART. When the equation obtained from the CNc was applied to the extended validation sample, the KART-predicted IQs of the AD, MCI and the CN groups did not significantly differ, whereas their current global cognition scores significantly differed between the groups. In conclusion, the results support the validity of KART-predicted IQ as an index of premorbid IQ in individuals with AD.

## Introduction

Obtaining a good estimate for an individual’s premorbid cognitive functioning is critical in research as well as in practice to reliably interpret current cognitive performance or identify cognitive declines. Given that most patients do not have records of their cognitive functioning prior to onset of complaints that led them to seek evaluations, an essential aspect of neuropsychological assessments is obtaining the estimates of premorbid intelligence [1]. The most common approach to estimating premorbid intelligence is the use of a word-reading test, which requires the participant to verbally pronounce orthographically irregular words in a language. It is assumed that correct pronunciation of these words implies prior knowledge of them and therefore a higher premorbid intelligence [2].

A variety of different word-reading tests in English have been developed over the years, with its own strengths and weaknesses. The first reading tests designed to estimate premorbid intelligence were the Schonell Graded Word Reading Test [3] and the National Adult Reading Test (NART) [4]. The NART, developed in England, has been adapted for American English speakers (*i*.*e*., AMNART) soon after the development of the NART [5]. Main rationale for such tests is that reading ability is shown to be resistant to mental deterioration due to organic causes [6]. Previous studies have demonstrated that performance on a word reading test is resistant to deterioration in patients with mild to moderate dementia of different etiologies and that its scores are stable after one year of progressive declines in other cognitive functions [7–10].

Following the NART, word-reading tests were developed in other languages such as the French adaptation of the NART (fNART) [11], the Word Accentuation Test (WAT) in Spanish [12], and the Japanese version of the NART (JART) [13]. To date, unfortunately, there are no similar tools developed or validated in Korean-speaking population.

English and Korean both employ alphabetic writing system. A notable difference is that Korean alphabets, *hangul*, are written in syllable blocks rather than a linear string of letters as in the Roman alphabetic writing system. *Hangul* presented in syllable blocks allows for less ambiguous phonological information compared to English with nontransparent grapheme-phoneme correspondences [14]. Despite simpler syllabification of Korean, relationship between orthography to phonology in Korean language is not always straightforward. More specifically, discrepancies between orthography and pronunciation exist from phonological transformations such as coda neutralization, double consonant liaisons, tensification, liquidation, and nasalization [15]. In Korean, as is the case with English, awareness of the such discrepancies when reading thereby producing correct pronunciation is a good inference for prior knowledge. Taking the differences between Korean and English into consideration, Korean adaptation of the word-reading test was carefully approached to incorporate comparable but unique characteristic of Korean language.

The current study aimed to develop a word-reading test for Korean-speaking adults using irregularly pronounced words that would be useful in estimation of premorbid intelligence. A carefully selected set of words for the Korean adult reading test (KART) was examined for its validity and reliability using cognitive normal (CN) elderly and patients diagnosed with Alzheimer’s disease dementia (AD) or amnestic mild cognitive impairment (MCI), which is known as a prodromal state of AD. In addition, regression equations were developed to estimate the Wechsler Adult Intelligence Scale, 4^th^ edition (WAIS-IV) intelligence quotients (IQ) from the KART scores and years of education.

## Methods and methods

### Participants

All participants—native Korean speakers—underwent comprehensive clinical and neuropsychological evaluations for diagnosis of CN, MCI, or AD prior to administration of the KART. Two independent participant samples were used to obtain two separate sets of data. The first set was used for development and construction of the KART while the second set was used for extension of the validation process. The first set used in the development phase included 60 CN elderly and 31 patients with AD. CN participants were recruited from community and AD patients were recruited from a pool of patients who visited the Dementia and Age-Associated Cognitive Decline Clinic of the Seoul National University Hospital in Seoul.

The second set of the participants used in the extended validation phase were recruited from the Korean brain aging study for early diagnosis and prediction of Alzheimer’s disease (KBASE), an ongoing prospective cohort study. Because characteristics that may have confounding effects on cognition differ substantially between the diagnostic groups, we used propensity score methods to generate more balanced groups that have similar observed characteristics such as simple demographic. Propensity scores are conditional probabilities of belonging to a particular group, given a set of observed background characteristics [16, 17]. We used age, education and gender in our propensity score matching model and 84 CN, 56 MCI, and 43 AD patients were included in the final second set.

The CN subjects did not have subjective or reported cognitive complaints; and, they had Clinical Dementia Rating (CDR) score of 0 [18], Mini-Mental State Examination (MMSE) score greater than or equal to 26 [19], and performance scores all within normal range based on the respective age-, education-, and gender-specific normative means of the tests included in the Korean version of the Consortium to Establish a Registry for Alzheimer’s Disease (CERAD-K) neuropsychological battery [20, 21]. Individuals with MCI met the Petersen’s criteria [22], which are: (a) memory complaint corroborated by an informant; (b) objective memory impairment for age, education, and gender; (c) essentially preserved general cognitive function; (d) largely intact functional activities; and (e) not demented. All aMCI individuals had an overall CDR of 0.5. In terms of the criterion (b), a performance score for at least one of the four episodic memory tests included in the Korean version of the Consortium to Establish a Registry for Alzheimer’s Disease (CERAD-K) neuropsychological battery (namely, the Word List Memory, the Word List Recall, the Word List Recognition, and the Constructional Recall test) [19, 21] was at least 1.5 standard deviation below the respective age-, education-, and gender-specific normative means [20]. Patients diagnosed with AD met the criteria for dementia according to the Diagnostic and Statistical manual of Mental Disorders, 4^th^ edition text revision (DSM-IV-TR) [23] as well as the criteria of probable AD of the National Institute of Neurological and Communicative Disorders and Stroke and the AD and related Disorder Association (NINCDS-ADRDA) [24] and had global CDR score of 0.5 or 1.

A panel of clinical experts in dementia research made decisions on clinical diagnoses and CDR for all participants after reviewing all available data. The exclusion criteria for participants were as follows: presence of any serious medical, neurological, or psychiatric disorders that could affect mental function and the absence of a reliable informant. All participants and their caregivers for dementia patients were given a complete description of the study prior to providing written informed consent to participate in this study, which was approved by the Institutional Review Board of the Seoul National University Hospital.

### Development and construction of the KART

#### Selection of words for the KART

A linguist who specialized in Korean phonology selected 94 words with irregular relationship between orthography to phonology. All participants were asked to read out loud the 94 words printed on 3 sheets of white paper (A4 size) and the test administers scored whether or not the participants pronounced the words correctly. All participants were also administered the full battery of the Korean version of the WAIS-IV (K-WAIS-IV) [25]. CN subjects were randomly divided into two groups: CN calculation (CNc) and CN validation (CNv). Among the 94 words, 50 words that did not show a significant difference between the CNc group and the dementia group were selected and constituted the KART.

#### Testing reliability of the KART

Internal consistency of the finally selected 50 words was calculated using Cronbach’s alpha coefficient (*α*) in all CN individuals. Eleven CN and 11 AD patients from the first set were randomly selected and retested by the same psychologist 4 weeks after the initial administration in order to assess test-retest reliability using the intra-class correlation coefficient (ICC *ρ*). Another 10 CN and 10 AD patients from the first set were randomly selected from the remaining participant pool to assess inter-rater reliability; audio-recordings of the participants’ responses were blindly scored by two psychologists and the Pearson correlation coefficient (Pearson’s *r*) was calculated. Number of correct responses of the 50 words was used to calculate the reliability coefficients.

#### Validation of the KART-predicted IQs

Linear regression analysis was conducted to generate equations to predict IQs using the KART error score and years of education in the CNc group. Then, goodness of fit of linear regression was assessed using the KART predicted IQs. Finally, the observed IQs based on the K-WAIS-IV and the KART-predicted IQs were compared between the CNv and AD groups in order to determine validity of the KART using independent samples *t*-tests.

### Extended validation of the KART

Using the second participant set, the KART-predicted IQs were calculated based on the equations derived from the development phase and were compared between CN, MCI, and AD using analysis of variance (ANOVA). In addition, the global cognition scores from the CERAD-K neuropsychological battery (*i*.*e*., CERAD total score I and total score II) [26] were compared between the groups.

### Other statistical analyses

Demographic data of the groups were compared using ANOVA for continuous variables and Chi-squared analysis (*χ*^*2*^-test) for categorical variable.

## Results

### Demographic characteristic

Demographics characteristics of the first set are reported in Table 1. Thirty CNc, 30 CNv, and 31 AD patients were compared on their age, gender distribution, and years of education. The AD group was older than the CNc or CNv groups; however, mean ages of the CNc and CNv groups did not significantly differ from each other. Gender distribution or mean years of education did not significantly differ between the groups.

**Table 1:** Demographic characteristics of the first set.

	CNc (*n* = 30)	CNv (*n* = 30)	AD (*n* = 31)	**Statistics**
Age (Mean(*SD*))	67.9(6.3)	68.4(5.9)	73.0(6.2)	*F(2*, *88)* = 6.45**
Female (%)	56.7	46.7	64.5	*χ*^*2*^ = 1.98
Education (Mean(*SD*))	12.0(4.0)	11.3(4.4)	11.5(3.9)	*F(2*, *88)* = 0.22

*Note*. CNc, cognitively normal calculation group; CNv, cognitively normal validation group; AD, Alzheimer’s disease dementia; SD, standard deviation.

**p < .01

Demographic characteristics of the second set are reported in Table 2. As expected, mean age, years of education and gender distribution of the AD and the MCI compared to CN did not yield significant differences (*p* = .81, *p* = .36, *p* = .68, respectively).

**Table 2:** Demographic characteristics of the second set.

	CN (*n* = 80)	MCI (*n* = 56)	AD (*n* = 43)	**Statistics**
Age (Mean(*SD*))	73.17(6.5)	73.13(6.7)	73.93(7.6)	*F(2*, *180) =* 0.21
Female (%)	64	71	67	*χ*^*2*^ = 0.78
Education (Mean(*SD*))	9.85(4.8)	10.64(4.6)	9.26(5.4)	*F(2*, *180)* = 1.03

*Note*. CN, cognitively normal; AD, Alzheimer’s disease dementia; aMCI, amnestic mild cognitive impairment; SD, standard deviation.

### Reliability

Internal consistency of the selected 50 words was acceptable (Cronbach’s *α* = 0.77). Test-retest reliability was strong (ICC *ρ* = 0.79, *p* < .001). Inter-rater reliability was also strong (Pearson’s *r* = 0.99, *p* < .001).

### KART-predicted IQs

Equations to predict IQs based on performance on the KART were developed. In order to employ the Grober and Sliwinski’s formula of the AMNART [5] that was validated using elderly subjects with dementia and has been shown to be a robust method in estimating premorbid intelligence in elderly, regression equations that include the KART error scores and years of education of the CNc group were derived to estimate the KART-predicted Full Scale IQ (KART-FSIQ), the KART-predicted Verbal Comprehension Index (KART-VCI), the KART-predicted Perceptual Reasoning Index (KART-PRI), the KART-predicted Working Memory Index (KART-WMI), and the KART-predicted Processing Speed Index (KART-PSI). The prediction equations are as follows:

KART-FSIQ = 113.28–1.45 x KART errors + 0.85 x Education

KART-VCI = 107.88–1.07 x KART errors + 1.33 x Education

KART-PRI = 116.56–1.30 x KART errors + 0.09 x Education

KART-WMI = 113.94–1.40 x KART errors + 0.53 x Education

KART-PSI = 106.58–0.86 x KART errors + 0.71 x Education

KART-predicted FSIQ, VCI, PRI, WMI, and PSI accounted for 63%, 48%, 25%, 46%, and 33% of the variances in observed WAIS-IV FSIQ, VCI, PRI, WMI, and PSI, respectively.

### Cross validation

The prediction equations were applied to the CNv group for validation. In order to determine if the size of residual with regression was related to IQ levels, correlation analyses between IQs and the size of the residual were performed. Approximately 97% (FSIQ, 30/30; VCI, 30/30; PRI, 29/30; WMI, 29/30; PSI, 30/30) of the group showed their standardized residuals (SR) within ± 2.00 and the SR did not have specific patterns relating to KART error scores. Standardized errors (SE) of the estimate of the KART-FSIQ, KART-VCI, KART-PRI, KART-WMI and KART-PSI were 9.51, 10.22, 10.35, 9.86, and 7.30, respectively.

**Fig 1:**
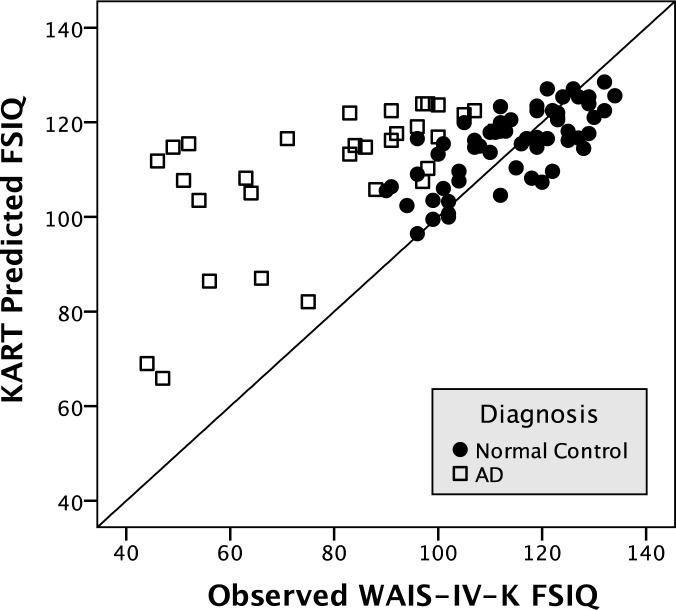
Distribution of Korean Adult Reading Test (KART)-predicted full-scale IQ (FSIQ) and observed FSIQ.

In the CNv group, all of the KART-predicted IQs were significantly correlated with the K-WAIS-IV observed IQs (FSIQ, *r* = 0.67, *p* < .001; VCI, *r* = 0.65, *p* < .001; PRI, *r* = 0.41, *p* < .05; WMI, *r* = 0.55, *p* < .01; PSI, *r* = 0.66, *p* < .001). The observed IQs were significantly different between the CNv and the AD group; however, the KART-predicted IQs were not significantly different between the groups (Table 3). When the equation was applied to the AD group, all of the subjects had higher KART predicted FSIQ than the observed FSIQ (Fig 1).

**Table 3:** Differences of K-WAIS-IV observed IQs and KART-predicted IQs between CNv and AD groups.

	**CNv**		**AD**		*t*	*p***-value**
	**Mean(SD)**	**Range**	**Mean(SD)**	**Range**		
KART-FSIQ	114.4(7.9)	100.0–128.5	109.3(15.5)	65.9–123.9	1.63	.11
Observed FSIQ	118(12.8)	90.0–132.0	78.9(21.1)	44.0–111.0	7.41	< .001
KART-VCI	116.7(8.5)	102.5–131.9	113.1(12.9)	79.5–127.1	1.27	.21
Observed VCI	114.4(13.9)	92.0–134.0	89.5(19.3)	59.0–120.0	5.78	< .001
KART-PRI	108.0(4.8)	96.2–115.5	103.2(12.7)	60.7–114.7	1.96	.06
Observed PRI	106.6(10.9)	86.0–126.0	80.5(21.1)	50.0–109.0	6.09	< .001
KART-WMI	111.8(6.6)	98.3–123.5	106.8(14.4)	63.8–120.0	1.75	.09
Observed WMI	110.3(11.7)	87.0–142.0	87.8(15.1)	52.0–115.0	6.50	< .001
KART-PSI	109.5(5.4)	100.7–119.3	106.6(9.6)	81.5–116.1	1.49	.14
Observed PSI	107.4(9.8)	92.0–125.0	75.3(19.9)	50.0–110.0	8.00	< .001

*Note*. CNv, cognitively normal validation group; AD, Alzheimer’s disease dementia; SD, standard deviation; KART, the Korean Adult Reading Test; K-WAIS-IV, Korean version of the Wechsler Adult Intelligence Scale, 4^th^ edition; KART-FSIQ, KART-predicted Full Scale IQ; KART-VCI, KART-predicted Verbal Comprehension Index; KART-PRI, KART-predicted Perceptual Reasoning Index; KART-WMI, KART-predicted Working Memory Index; KART-PSI, KART-predicted Processing Speed Index.

### Extended validation of the KART

The equations were applied to the second set and compared between the groups. The KART-predicted IQs of the AD, MCI and CN groups did not significantly differ; however, the current global cognition scores (*i*.*e*., CERAD-K TS I & II) significantly differed between the groups (Table 4).

**Table 4:** Differences of CERAD-K total score (TS) and KART-predicted IQs between the groups.

	**CN**		**MCI**		**AD**		*F*	*p-***value**
	**Mean(SD)**	**Range**	**Mean(SD)**	**Range**	**Mean(SD)**	**Range**		
KART-FSIQ	111.26(12.5)	71.2–127.4	110.78(12.0)	67.6–123.0	107.83(15.0)	64.0–125.4	1.04	.35
KART-VCI	113.31(12.0)	76.9–132.3	113.51(11.4)	78.4–129.1	110.36(14.2)	71.5–128.1	0.99	.37
KART-PRI	108.13(9.0)	78.9–118.0	107.16(8.8)	71.6–116.3	105.45(11.0)	72.4–117.4	1.14	.32
KART-WMI	109.12(11.0)	73.3–122.4	108.43(10.7)	68.1–118.9	105.99(13.4)	66.3–121.0	1.08	.34
KART-PSI	107.41(8.2)	81.6–119.1	107.29(7.8)	80.7–116.5	105.25(9.8)	77.3–117.1	1.02	.36
CERAD-K TS I	69.60(10.6)	46–95	52.04(10.7)	30–71	43.21(10.2)	21–63	102.62	< .001
CERAD-K TS II	76.24(12.3)	47–105	54.41(11.8)	30–78	44.14(10.6)	23–65	112.32	< .001

*Note*. CN, cognitively normal; AD, Alzheimer’s disease dementia; aMCI, amnestic mild cognitive impairment; SD, standard deviation; KART, the Korean Adult Reading Test; KART-FSIQ, KART-predicted Full Scale IQ; KART-VCI, KART-predicted Verbal Comprehension Index; KART-PRI, KART-predicted Perceptual Reasoning Index; KART-WMI, KART-predicted Working Memory Index; KART-PSI, KART-predicted Processing Speed Index; CERAD-K TS I, CERAD-K total score I; CERAD-K TS II, CERAD-K total score II.

In CN, all of KART-predicted IQs were significantly correlated with the current global cognition scores (*i*.*e*., Pearson’s correlations with CERAD-K TS 1; KART-FSIQ: *r* = .38, *p* < .001; KART-VCI: *r* = .36, *p* < .001; KART-PRI; *r* = .38, *p* < .001; KART-WMI: *r* = .38, *p* < .001; KART-PSI: *r* = .37, *p* < .001).

## Discussion

The KART showed acceptable internal consistency and had excellent reliability. In terms of the validity of the KART, the linear regression equations developed using the calculation group (*i*.*e*., CNc) were well-fitted to the validation group (*i*.*e*., CNv). Furthermore, the K-WAIS-IV IQs had high and significant correlations with the KART-predicted IQs in the CNv group. Additionally, while the observed K-WAIS-IV IQs were significantly different between the CNv and AD groups, in that the mean IQs of the AD group was significantly lower than that of the CNv group, the KART-predicted IQs were very similar between the two groups. These results support the validity of the KART-predicted IQ as an index of premorbid IQ in individuals with AD.

The extended validation process using the separate participant dataset further supported validity of the KART. First, intelligence scores derived from the regression equations using the KART scores and years of education are strongly associated with current cognitive functioning in CN elderly but not in individuals with MCI and AD. Second, the KART-derived intelligence scores are unaffected by the current cognitive impairment, as evidenced in the MCI and AD groups. These findings further support the utility of the KART to estimate premorbid intelligence in individuals with MCI and AD.

The regression equations derived in this study include the number of KART errors and years of education. Similar to the previous studies [5, 12], the equations that used the KART errors and education and those with the KART errors alone were only slightly different. More specifically, inclusion of education as a predictor slightly reduced the standard error of the estimate (6.40 versus 6.97) and produced a small increase in the correlations (0.618 versus 0.614) between the KART-predicted IQs and the observed WAIS-IV IQs. The ranges of possible predicted IQs from the equations with the KART errors and years of education as predictors were similar to the ranges of the predicted IQs using only the KART errors. Previous studies vary on whether to include years of education in the equations or not and there are no clear consensus due to unique characteristics of the participant populations. For this study, given slightly increased accuracy, only the equations using both the KART errors and education are reported.

The correlations between the observed IQs and the KART-predicted IQs from the current study are analogous to those reported from other word-reading tests developed in different languages [5, 12, 13]; more specifically, the correlations between the observed IQ and the KART-, AMNART-, JART-, and WAT-R-predicted premorbid intelligence were 0.67, 0.73~0.79, 0.88, and 0.82, respectively. Slight differences in correlation coefficients may partly be due to presence of dialects/vernaculars in Korean language based on regions of where each participant was raised. However, the effects of vernaculars on either the estimates of intelligence or the degree of correct reading of irregularly pronounced words have not been studied previously and the relationships are unclear. Further clarification can be offered through an extension of the study by increasing the sample size including only the participants whose education was not affected by the Korean War during which time individuals were unable to benefit from the standard education curriculum that included uniform reading lessons.

Some caution must be used when utilizing the KART. First, similar to the previous studies with NART [7, 10, 27], our data showed that the KART was relatively poor in predicting performance IQ. Thus, users must be cautious when using the KART to estimate premorbid performance IQ. Second, as with the other studies that used regression procedures, the range of possible predicted IQs is limiting compared to the observed WAIS-IV IQs; therefore, the KART-predicted IQs for individuals with high intelligence would likely be underestimated.

## Conclusions

This is the first study to develop and derive a tool to estimate premorbid intelligence of Korean speaking adults. It provides a way to estimate IQs from the information obtained quickly and reliably. Obtaining premorbid IQ in those with dementia, AD in particular, or cognitive impairments has obvious potentials in clinical settings. Notable deviation of observed cognitive functioning from estimated premorbid intellectual functioning can indicate the extent to which cognitive impairment has progressed or severity of the deterioration. Although the present results showed that the reading of irregular words in Korean was well-preserved in mild AD, as with the NART for native English speakers, further study is needed to determine how the model is affected by the disease severity and/or language impairment.
